# Targeted Next-Generation Sequencing in Drug-Resistant Tuberculosis: WHO Guidance and Practical Implementation Priorities

**DOI:** 10.3390/biomedicines14010093

**Published:** 2026-01-02

**Authors:** Sungwon Jung

**Affiliations:** 1 Department of Genome Medicine and Science, Gachon University College of Medicine, Incheon 21565, Republic of Korea; sjung@gachon.ac.kr; Tel.: +82-32-458-2740; 2 Gachon Institute of Genome Medicine and Science, Gachon University Gil Medical Center, Incheon 21565, Republic of Korea

**Keywords:** tuberculosis, drug resistance, targeted NGS

## Abstract

Targeted next-generation sequencing (tNGS) closes the gap between point-of-care rapid tests and phenotypic drug susceptibility testing (pDST) in drug-resistant tuberculosis (DR-TB). The 2025 World Health Organization (WHO) consolidated guidelines and the operational handbook place tNGS after initial automated nucleic acid amplification tests (aNAATs) for the delivery of catalogue-linked molecular drug susceptibility testing (DST) for a broad drug panel, reserving whole-genome sequencing (WGS) and/or pDST for discordance resolution, confirmation, and surveillance. This review summarizes (i) the core tNGS principles and panel design; (ii) platform-specific workflows for Illumina and Nanopore, including direct-from-sample implementations and typical turnaround times; (iii) catalogue-based interpretation against the 2023 WHO *Mycobacterium tuberculosis* mutation catalogue, with emphasis on bedaquiline/clofazimine (BDQ/CFZ) resistance and management of uncertain variants; (iv) pooled accuracy and sources of genotype–phenotype discordance; and (v) practical requirements for bioinformatics, quality assurance/external quality assessment (QA/EQA), and standardized reporting. We summarize operational and economic considerations (throughput, batching, and network design) to clarify where tNGS adds value compared with alternative strategies and to outline priority research needs, including (i) performance standards for culture-free tNGS, (ii) robust heteroresistance detection, (iii) standardized variant curation, and (iv) data-sharing frameworks to refine genotype–phenotype links. When embedded within validated QA/EQA frameworks and catalogue-linked reporting systems, tNGS can shorten the time to effective therapy by rapidly informing fluoroquinolone (FQ) susceptibility and providing early, tiered resistance signals for newer agents (e.g., BDQ), with indeterminate findings prompting reflex pDST/WGS.

## 1. Introduction

Tuberculosis (TB) remains one of the leading causes of death from infectious disease. In 2023, an estimated 10.8 million people developed TB and 1.25 million died, highlighting the need to shorten the time to effective therapy and expand access to timely, comprehensive drug-resistance results [1,2]. Commonly used TB diagnostic modalities include smear microscopy, aNAATs (e.g., GeneXpert^®^ (Cepheid, Sunnyvale, CA, USA)), line probe assays, and culture-based methods, including pDST, each providing different trade-offs in speed, scope, and operational requirements. Although aNAATs have improved early detection, comprehensive resistance profiling remains a bottleneck. Culture-based pDST typically takes ≥2 weeks and diagnostic workflows are often fragmented across sequential assays [3].

In response, the WHO consolidated guidelines and the updated operational handbook retain tNGS as a recommended approach and clarify its placement in the diagnostic algorithm as follows: (1) aNAATs for initial detection, (2) tNGS for broad, rapid, catalogue-linked molecular DST, and (3) WGS or pDST for confirmation of discordant/indeterminate results and for surveillance. They also permit testing from culture DNA or validated direct clinical specimens, set QA/EQA expectations, and standardize catalogue-based interpretation and reporting so outputs inform programmatic decisions without prescribing treatment [4,5]. While the WHO guidelines and operational handbooks define the normative role of tNGS in the diagnostic cascade, they do not provide an end-to-end implementation blueprint. Here, we synthesize the evidence into practical implementation priorities—linking catalogue tier interpretation to callability/VAF rules, QA/EQA governance, and reflex testing—and provide an actionable specimen-to-report checklist alongside operational and economic network considerations. In WHO operational materials, multiple tNGS solutions for TB drug-resistance detection are referenced (e.g., Deeplex^®^ Myc-TB (GenoScreen, Lille, France), AmPORE-TB^®^ (Oxford Nanopore Technologies, Oxford, UK), and TBseq^®^ (Shengting Medical Technology, Hangzhou, China)). Mention of specific products in this review is for identification and implementation discussion and does not imply endorsement.

Operationally, the pathway is as follows: (1) aNAATs at or near the point of care to confirm TB and screen sentinel resistance, (2) tNGS to generate a broad molecular resistance profile via catalogue-linked interpretation, and (3) WGS or pDST when results are discordant, indeterminate, or needed for surveillance (pDST typically adds ≥2 weeks to turnaround) [3,4,5]. This staging prioritizes speed for initial therapeutic decisions while preserving confirmatory and public-health functions downstream.

tNGS closes the coverage–speed gap between rapid triage tests (aNAATs and line probe assays (LPAs)) and culture-dependent phenotypic testing by interrogating dozens of resistance-associated loci in a single assay [4,5]. It delivers actionable, broad resistance profiling on a near-same-day timescale, including markers relevant to next-generation regimens. WGS provides maximal genomic scope for novel-mechanism discovery, high-resolution surveillance, lineage assignment, and troubleshooting, while pDST remains the phenotypic reference standard/arbiter when genotype–phenotype links are incomplete [3,4,5].

To translate results into policy-aligned choices, reports should provide per-drug calls with evidence levels and variant notes. Predicted fluoroquinolone susceptibility with no BDQ resistance signals may preserve eligibility for short all-oral options where program policy permits, whereas predicted resistance or indeterminate calls trigger alternative pathways and confirmatory WGS or pDST rather than empiric exposure. Because tNGS shortens turnaround relative to pDST, it advances the decision window by days to weeks, particularly in drug-resistant TB admissions and decentralized network settings [3,4,5].

Clinical actionability depends on robust program governance. Bioinformatics pipelines must be validated and version-controlled with audit trails from raw reads to the final report. Challenges remain, including heteroresistance and mixed infection, unresolved technical challenges in uncommon specimen types and low-bacillary-load samples, and our incomplete knowledge of resistance-related mutation catalogues. As diagnostic pathways mature with consideration of currently available technical benefits as well as inherent limitations, tNGS can more reliably shorten the time to effective therapy while preserving surveillance and stewardship safeguards within the WHO 2025 cascade [4,5].

## 2. Principles and Panel Design: From Multiplex Amplification to Catalogue-Based Interpretation

**Figure 1:**
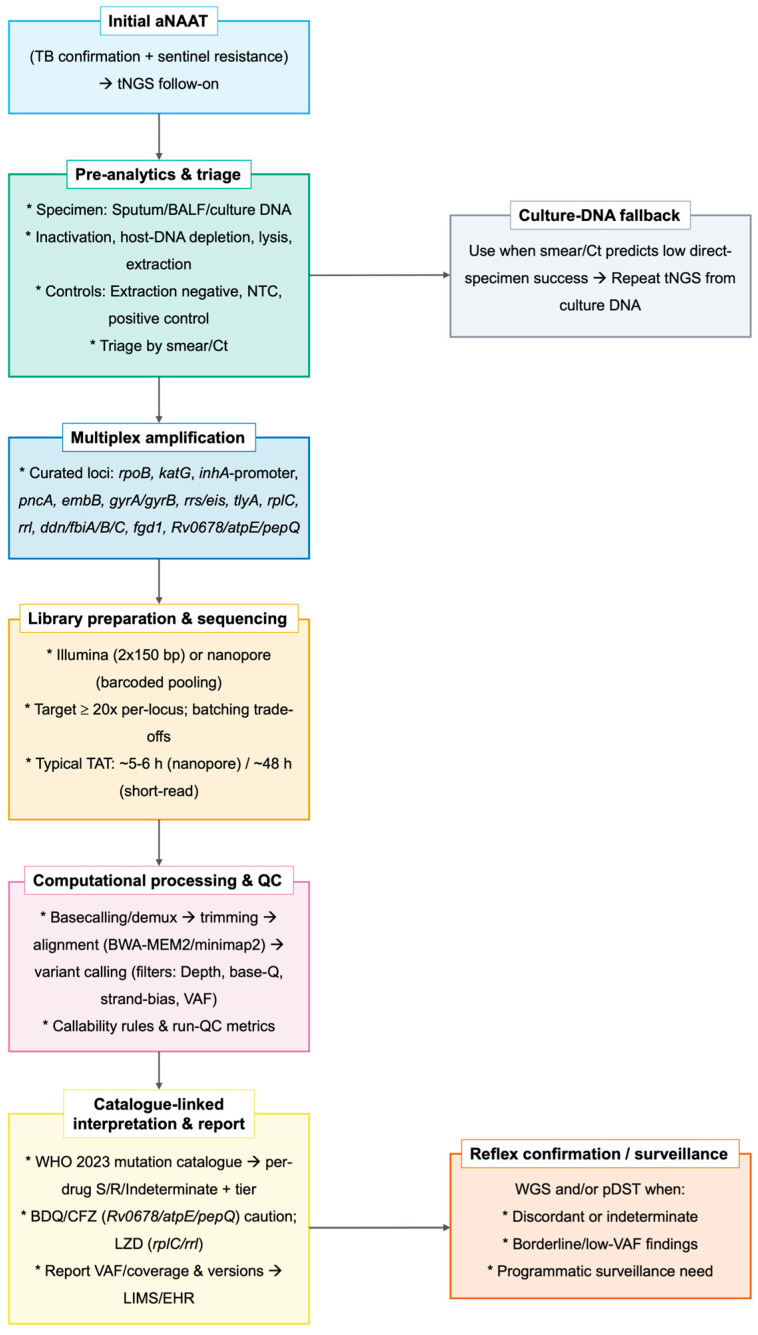
Specimen-to-report pipeline for tNGS in DR-TB within the WHO 2025 diagnostic cascade. After the aNAAT confirms TB and sentinel resistance, specimens undergo standardized pre-analytics and triage. Curated multiplex amplicons cover key resistance loci. Libraries are sequenced on short-read or nanopore platforms. A version-locked pipeline applies QC/callability rules and VAF thresholds before catalogue-linked interpretation against the 2023 WHO *M. tuberculosis* mutation catalogue to produce per-drug calls (susceptible(S)/resistant(R)/indeterminate) with confidence tiers. Reports disclose coverage/VAF thresholds and software/catalogue versions for auditability and laboratory information management system (LIMS)/electronic health record (EHR) integration. Discordant or indeterminate results trigger reflex WGS and/or pDST; QA/EQA governance spans these steps.

tNGS for DR-TB can be implemented as a reproducible specimen-to-report pipeline: (1) the multiplex amplification of curated resistance loci; (2) library preparation and sequencing on the Illumina or Nanopore platform; (3) read-level QC and alignment; (4) variant calling with assay-specific depth and variant allele frequency (VAF) filters; and (5) the catalogue-based translation of variants into per-drug calls with an auditable report [4,5,6]. In the 2025 WHO pathway, this workflow follows initial aNAATs and accepts DNA from cultures or validated direct clinical specimens, with QA/EQA and transparent version control required for cross-site reproducibility [4,5]. Figure 1 summarizes the amplification, sequencing, analytics, catalogue-linked interpretation, and standardized reporting in a single process map [4,5,6].

### 2.1. Panel Design Linked to Therapy

Panels should directly inform treatment by combining hotspot and full-gene coverage for first-generation, second-generation, and newer drugs [4,5,6,7]. Typical inclusions: *rpoB* (rifampicin resistance-determining region (RRDR); rifamycins), *katG* and *inhA* promoter (±*ethA/ndh*) for isoniazid/ethionamide; full-gene *pncA* for pyrazinamide; *embB* (e.g., M306) for ethambutol; *gyrA/gyrB* quinolone resistance-determining regions (QRDRs) for FQs; *rrs* (A1401G/G1484T) and *eis* promoter for amikacin/kanamycin; *tlyA* for capreomycin, linezolid (LZD) markers (*rplC*, 23S rRNA/*rrl*), nitroimidazoles (*ddn, fgd1, fbiA/B/C/D*), and BDQ/CFZ loci (*Rv0678*, *atpE*, *pepQ*) [6,7,8]. Designs should future-proof GC-rich/variable regions with tiling, avoid lineage-specific single-nucleotide polymorphisms (SNPs) at primer sites (to prevent lineage-dependent amplicon dropout), prefer full-gene coverage where spectra are diffuse (e.g., *pncA*, *Rv0678*), and be version-linked to the evolving WHO mutation catalogue [5,6,7]. Conversely, assays confined to narrow hotspot coverage or prone to primer dropout risk sensitivity loss and drug-specific no-calls, particularly where resistance-associated variants occur outside canonical hotspots or are dispersed across the gene.

### 2.2. Primer/Amplicon Design and Controls

Robustness depends on primer sets that tolerate GC-rich loci and lineage diversity. Recommended practices include (i) redundant tiling with platform-appropriate amplicons, (ii) the in silico screening of primer sites against the Mycobacterium tuberculosis complex (MTBC) and common nontuberculous mycobacteria (NTM), (iii) optimizing the melting temperature (Tm) and GC content to minimize primer–dimer formation, and (iv) the empirical rebalancing of pools after pilot runs [4,5,7,9,10,11,12]. Illumina favors shorter, uniform targets, whereas Nanopore tolerates longer amplicons and can span high-GC stretches (e.g., Deeplex^®^ Myc-TB; AmPORE-TB^®^) [9,11,13,14]. To mitigate carry-over contamination, deoxyuridine triphosphate (dUTP)/uracil-DNA glycosylase (UNG) is used and no-template, positive, and internal controls per batch are included [4,5,10]. Primer sets should be sequence-documented, LIMS-tracked, and change-controlled, and tools like Primer3 should be used to assist constraint checking pre-validation [5,7,12].

### 2.3. Library Preparation, Depth, and Turnaround Time

Illumina panels (e.g., Deeplex^®^ Myc-TB) commonly use paired-end 2× 150 bp with flexible indexing for throughput–turnaround time (TAT) trade-offs and support DNA from inactivated specimens or cultures with validated input ranges (≈10^2^–10^3^ genomes/test) [9]. Nanopore workflows (e.g., AmPORE-TB^®^) barcode and pool amplicons; typical batches (≈22 samples plus controls) can deliver same-day results, with a recommended median per-locus coverage of ≥20× [13,15]. High on-target depth underpins detection of minor variants/heteroresistance and often exceeds culture-WGS at resistance loci [9]. Although not currently required in TB tNGS kits, unique molecular identifiers (UMIs)/duplex tagging can suppress ultra-low-frequency errors, albeit at the cost of added workflow complexity [16,17]. All workflows should conform to WHO guidance on QA/EQA and reportable ranges [5].

### 2.4. Computational Processing

A version-locked pipeline typically includes basecalling and demultiplexing (for Nanopore), adapter/primer trimming, alignment, variant calling with prespecified filters, and catalogue-based interpretation [5,6]. Common tools include BWA-MEM2 for short reads and minimap2 for long reads; Cutadapt and iVar help prevent primer-proximal artefacts [18,19,20,21]. Oxford Nanopore Technologies (ONT; Oxford, UK) amplicon workflows (e.g., wf-amplicon) pair minimap2 mapping with Medaka polishing and advise depth normalization for stable single-nucleotide variant (SNV)/indel calls [22]. Filters should specify the per-locus minimum depth, base quality, strand bias metrics, and VAF thresholds; kits may raise thresholds at known noisy sites and report subclonal calls separately [9]. Final calls are mapped to the WHO mutation catalogue with transparent evidence tiers and exported via validated tools (e.g., TB-Profiler, Mykrobe, SAM-TB) for standardized genotype–phenotype reporting [5,23,24,25].

### 2.5. Catalogue-Based Interpretation and Reporting

The WHO catalogue assigns variants as associated with resistance, not associated, or uncertain. The decision logic is as follows: (i) presence of a resistance-associated variant in a relevant gene ⟶ resistant; (ii) no such variant with adequate coverage ⟶ susceptible; (iii) uncertain variants or inadequate coverage ⟶ indeterminate, with reflex to WGS/pDST as appropriate [4,5,6]. When multiple variants co-occur, the highest-risk call and document conflicts should be prioritized (e.g., *Rv0678* heterogeneity for BDQ/CFZ) [6,8]. Reports should follow a template-mapping gene ⟶ variant (H37Rv coordinate, amino acid change) ⟶ catalogue tier ⟶ drug call, and include a brief narrative/action text summarizing the key calls, caveats (e.g., no-call/indeterminate due to coverage/VAF limits), and recommended follow-up, along with coverage metrics and software/catalogue versions; TB-Profiler/Mykrobe/SAM-TB can automate this mapping and formatting [5,23,24,25].

### 2.6. Direct-from-Sample (Culture-Free) tNGS

Success depends on pre-analytics. Acceptable types include sputum and, where validated, bronchoalveolar lavage fluid (BALF)/other respiratory specimens; standard inactivation and NALC-NaOH processing precede extraction [5,7,26]. Because host DNA can overwhelm the mycobacterial signal, many workflows add host depletion (e.g., saponin + DNase I) [27,28]. Mechanical/enzymatic lysis (bead beating + lysozyme/proteinase K) improves recovery from the lipid-rich cell wall. Extraction controls and inhibition monitors flag failed or contaminated preparations. Feasibility should be triaged using smear-grade or aNAAT Ct values (practical proxies for bacillary loads), as the bacillary load strongly predicts the on-target depth and downstream locus callability in culture-free tNGS [5,26]. Published implementations (e.g., AmPORE-TB^®^) show same-day sputum sequencing with catalogue-interpreted resistance when pre-analytics are optimized [13,15].

### 2.7. Analytical Sensitivity, Heteroresistance, and Governance

The limit of detection (LoD) and limit of quantitation (LoQ) should be established with dilution-series controls and defined per locus, acknowledging the depth variability across GC-rich targets; loci failing the minimum depth/quality default to indeterminate rather than inferred susceptibility [4,5]. Minor-variant detection requires validated VAF thresholds plus strand bias/quality filters; laboratories should document the lowest verified VAF per locus and flag subclonal findings that could affect regimen eligibility [4,5,6,7,9]. In direct-from-sample workflows, an effective LoD depends on the bacillary load, host DNA depletion, and extraction efficiency; the per-sample on-target yield should be reported [5,7,13,15,26,27,28]. Low-coverage, borderline VAFs or clinical/epidemiologic discordance should prompt WGS and/or pDST rather than over-interpretation [4,5,6]. The LoD/LoQ, VAF cut-offs, and pipeline parameters must be version-controlled, verified via EQA/proficiency testing, and included—along with software/catalogue versions—in the report to ensure auditability and reproducibility [5,29].

## 3. Comparing Commercial and Emerging tNGS Workflows (Design, Turnaround, and Reporting)

We compare end-to-end tNGS workflows across pre-analytics → wet lab → sequencing → bioinformatics → interpretation/reporting, aligned with WHO 2025 guidance and operational standards [4,5,7]. Comparison categories include (a) panel content/coverage; (b) specimen inputs; (c) batching/throughput/TAT; (d) per-locus depth and minor-variant policies; (e) analytics stacks and catalogue linkage; (f) output format; (g) QA/EQA and LIMS integration; and (h) infrastructure and deployment requirements. We apply these to Deeplex^®^ Myc-TB (Illumina amplicon) [9,14,30] and AmPORE-TB^®^ (Nanopore amplicon) [11,13,15], citing peer-reviewed performance where available [13]. WHO guidance also references additional tNGS tests (e.g., TBseq^®^), and WHO evidence summaries include other solutions; therefore, the two workflows below are presented as illustrative end-to-end examples to ground implementation trade-offs and not as a comprehensive survey or a ranking/endorsement. Table 1 provides a side-by-side comparison summary of Deeplex^®^ Myc-TB and AmPORE-TB^®^.

### 3.1. Deeplex^®^ Myc-TB

Deeplex^®^ employs an Illumina amplicon architecture with multiplex primer pools, sample indexing, and benchtop sequencing for flexible batching while preserving locus-level depth [9,14,30]. Inputs include culture DNA and, where locally validated, inactivated clinical extracts; the vendor reports end-to-end cycle of ≤48 h from extracted DNA [9,14,30]. A vendor portal aligns reads, calls SNVs/indels, and generates per-drug predictions using a curated knowledge base. Reports include susceptible/resistant/indeterminate calls, variant coordinates and amino acid changes, per-locus coverage/QC, run identifiers, and human-readable plus tabular exports for LIMS [9,14,30].

### 3.2. AmPORE-TB^®^

AmPORE-TB^®^ implements a Nanopore amplicon strategy engineered for both direct-from-sample and culture inputs, using the rapid barcoding and pooling of ~22 samples (plus controls) per run. From extracted DNA, the TAT is ~5–6 h, enabling same-day decisions; peer-reviewed evaluations demonstrate sputum-based feasibility within this timeframe [11,13,15]. Achieving ≥20× median per-locus coverage is recommended (extend runtime if needed), with pre-analytics triaged by smear grade or aNAAT Ct and standardized upstream handling [13,15]. A portal-based pipeline performs species confirmation, lineage assignment, and resistance calling, applies locus-specific depth/quality thresholds, summarizes gene-level coverage, flags sample issues, and returns both human-readable and tabular outputs compatible with LIMS [11,13,15].

### 3.3. Panel Content and Coverage Differences

Both assays cover core first- and second-line loci (e.g., *rpoB, katG/inhA promoter ± fabG1/ethA, pncA, embB, gyrA/gyrB, rrs/eis, tlyA, rplC/rrl*) and incorporate species and lineage features within the same run [4,5,7,9,11,13,14,15]. Deeplex^®^ concentrates on 18 resistance-associated gene targets (within a 24-plex amplicon mix), using full-gene tiling at highly heterogeneous loci (e.g., *pncA, Rv0678, tlyA*) [9,14]. AmPORE-TB^®^ targets 24 resistance genes (within enrichment of ~27 gene targets, including additional species/lineage/QC targets), explicitly adds nitroimidazole pathway loci (*ddn, fbiA/B/C, fgd1*), and includes *atpE* alongside *Rv0678* for BDQ/CFZ, enabling predictions for delamanid/pretomanid and broader BDQ/CFZ inference [11,15]. Because the overlap and tiling depth vary by product and version, procedures should cite the specific kit version used [4,5,7,9,11,14].

### 3.4. Performance, LoD/LoQ, and Quality Systems

Both workflows yield actionable per-drug calls when locus-level depth and quality thresholds are met; the LoD/LoQ should be defined per locus, with shortfalls reported as indeterminate rather than inferred susceptibility [4,5]. For direct-from-sample use, AmPORE-TB^®^ shows same-day feasibility with ≥20× on-target coverage guidance [13,15], while Deeplex^®^ emphasizes uniform GC-rich coverage via tiling and assay-specific VAF thresholds to balance heteroresistance detection against noise [9,14]. Discordant or uncertain findings are routed to WGS or pDST per WHO Module 3 [4,5]. Programs are expected to participate in EQA and maintain LIMS traceability, run-level QC dashboards, and version-lock software and catalogues to ensure reproducibility and auditability [4,5,7].

### 3.5. Pros/Cons and Fit-for-Purpose Suggestion

Deeplex^®^ Myc-TB prioritizes throughput and short-read accuracy, with a vendor-reported ≤48 h cycle and a mature web app, which is well suited to centralized labs that batch samples and value panel stability [9,14,30]. AmPORE-TB^®^ optimizes for speed and culture-free feasibility, delivering same-day (~5–6 h) results in ~22-sample batches, which is ideal for rapid triage or decentralized procedures but more sensitive to pre-analytics and run-time depth management [11,13,15]. Both produce catalogue-interpreted per-drug calls and belong within the WHO aNAAT → tNGS → WGS/pDST cascade; indeterminate or discordant outputs should be escalated rather than over-interpreted [4,5]. Choice depends on use case: if time-to-result and direct-from-sample capabilities dominate, AmPORE-TB^®^ fits, while if high-throughput, short-read robustness and established LIMS integration is a higher priority, Deeplex^®^ may be better suited [4,5,7].

## 4. Interpreting Variants with the WHO Mutation Catalogue: Evidence Tiers and the BDQ/CFZ Axis

The WHO 2023 mutation catalogue is the global reference that links MTBC variants to drug-resistance phenotypes, enabling laboratories to convert tNGS/WGS findings into standardized per-drug calls with explicit confidence labels across first-generation, second-generation, and newer drugs (SNVs and short indels in canonical genes) [4,5,6]. The WHO’s 2025 guidelines and operational handbook require catalogue-linked reporting and disclosure of the confidence tier supporting each call [4,5].

The catalogue’s evidence framework uses matched genomic–phenotypic datasets and classifies mutations into five confidence groups, from *associated with resistance* (Groups 1 and 2) through *uncertain significance* (Group 3) to *not associated* (Groups 4 and 5), based on quantitative criteria (e.g., odds ratios, positive predictive values, FDR-corrected *p*-values) and predefined expert rules. The 2023 update expanded the evidence base, strengthening labels for BDQ, LZD, and delamanid [6,31,32].

Translating sequence findings to drug-level calls follows catalogue-linked logic as follows: First, verify adequate coverage/quality for all drug-relevant loci; any unmet locus defaults to indeterminate rather than inferring susceptibility [4,5,6]. Where coverage is adequate: (i) any variant classified *associated with resistance* for that drug (Groups 1 or 2) → resistant, (ii) only variants classified *not associated* (Groups 4 and 5) and complete locus coverage → susceptible, (iii) any uncertain-tier variant (Group 3) or borderline VAF below validated thresholds → indeterminate, prompting follow-up WGS or pDST as needed [4,5,6]. For multiple variants in the same pathway (e.g., *katG* plus *inhA promoter*), prioritize the highest-risk outcome and document conflicts, following catalogue definitions to avoid over-calling [4,5,6,32].

**Figure 2:**
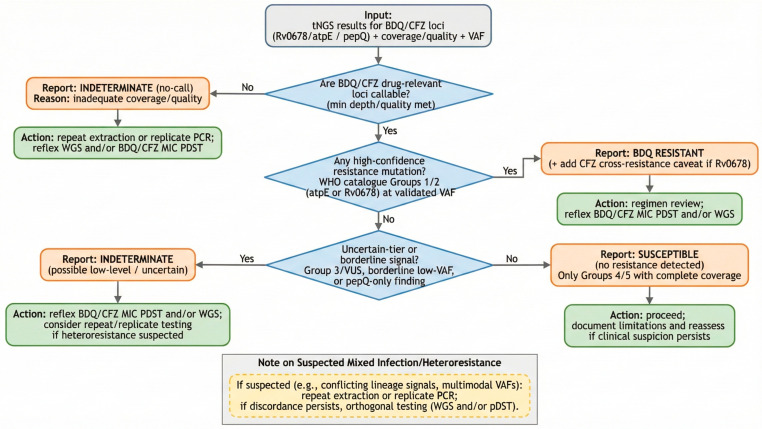
Simplified catalogue-linked decision algorithm for BDQ/CFZ interpretation in culture-free tNGS. The flowchart prioritizes locus callability (minimum coverage/quality) and validated VAF thresholds before assigning drug calls. High-confidence WHO mutation catalogue variants (Groups 1/2) in *atpE* or *Rv0678* support a BDQ-resistant call (with an explicit CFZ cross-resistance caveat for *Rv0678*), whereas uncertain-tier variants, borderline low-VAF signals, *pepQ*-only findings, or inadequate coverage default to indeterminate/no-call with reflex WGS and/or BDQ/CFZ MIC pDST. Where mixed infection or heteroresistance is suspected, repeat extraction or replicate PCR is recommended, with orthogonal testing if discordance persists.

Figure 2 provides a simplified, catalogue-linked decision algorithm for BDQ/CFZ interpretation, including callability/VAF prerequisites and reflex testing triggers. For BDQ and CFZ, the resistance biology centers on three loci: *Rv0678* encodes a repressor of the *MmpL5-MmpS5* efflux pump, where loss-of-function or regulatory changes derepress efflux, raising minimum inhibitory concentrations (MICs) to both BDQ and CFZ and producing cross-resistance with heterogeneous effect sizes [8,31,33,34]. *atpE*, encoding the c-subunit of ATP synthase (the BDQ target), harbors substitutions that yield high-level BDQ resistance but occur less frequently than *Rv0678* changes [6,8,35]. *pepQ* contributes as an accessory locus with context-dependent, generally more modest shifts [6,8]. The catalogue captures these relations via evidence tiers, emphasizing that many *Rv0678* variants remain uncertain and should not be over-called without supportive evidence [4,5,6].

Interpreting *Rv0678* requires a controlled approach. The variant spectrum is polytypic (missense, nonsense, frameshifts, in-frame indels, and regulatory changes) and dispersed across the gene, yielding heterogeneous MIC shifts and frequent BDQ ↔ CFZ cross-resistance [8,33,34]. A tentative best practice can be as follows: (i) call resistance only when the specific variant is catalogue-listed as associated with resistance, (ii) return indeterminate for uncertain-tier variants, borderline VAF signals, or inadequate coverage (must include explicit reflex WGS and/or BDQ/CFZ MIC pDST), and (iii) inclusion of a cross-resistance caveat in the narrative even when the final call is indeterminate [4,5,6,8,33,34].

By contrast, *atpE* substitutions, though rarer, are high-impact and often map to high-confidence tiers. When present and catalogued as associated, they support a BDQ-resistant call even with wild-type *Rv0678* [6,8,35]. *pepQ*-only findings typically remain uncertain and should default to indeterminate (with a narrative note on possible low-level effects and follow-up testing), while co-occurrence with catalogued *Rv0678* or *atpE* resistance variants should be documented but not double-counted in the per-drug call [4,5,6,8].

Uncertainty management on the BDQ/CFZ axis depends on the disciplined handling of subclonal variants and mixed populations. It is recommended to prespecify VAF thresholds and locus-level depth/quality rules so that variants below the validated VAF/LoQ or at loci failing coverage must not drive a resistant call and should be reported indeterminate with subsequent WGS or pDST [4,5,6]. Because *Rv0678* changes can emerge under drug pressure and produce heteroresistance, procedures should (i) avoid gene-level assumptions, (ii) disclose the exact variant and catalogue tier, and (iii) include a cross-resistance caveat for BDQ ↔ CFZ when *Rv0678* is implicated [6,8,31,32]. Suspicion of mixed infection (e.g., conflicting lineage signals, multimodal VAFs) warrants repeat extraction or replicate PCR and, if discordance persists, orthogonal testing (e.g., WGS and/or pDST) rather than over-interpretation [4,5].

Finally, reports should list, per drug, the gene → variant → catalogue tier → call, with coverage/QC metrics, observed VAFs and validated thresholds, and explicit pass/fail flags for loci that miss the minimum depth or quality [4,5,6]. For any drug-relevant locus that does not meet the minimum depth/quality thresholds, the result should be reported as no-call/indeterminate, and susceptibility should not be inferred from missing data. Governance requires version-controlled wet-lab and bioinformatics SOPs; LIMS traceability; change control and re-analysis triggers on catalogue updates; disclosure of software/reference database/catalogue versions and pipeline build/date; and routine EQA/proficiency participation to ensure inter-laboratory comparability [4,5,29].

## 5. Accuracy and Performance: Meta-Analysis and Field Evaluation

Accuracy is assessed per drug and specimen against a prespecified reference—pDST (MIC or critical concentration), WGS-based expert consensus, or a composite standard. Core metrics are the sensitivity (true positive (TP)/(TP + false negative (FN))), specificity (true negative (TN)/(TN + false positive (FP))), positive predictive value (PPV) and negative predictive value (NPV) (prevalence-dependent), and overall percent agreement (OPA). When the reference is imperfect or discordance is anticipated, the positive/negative percent agreement (PPA/NPA) is also reported. The operational performance uses an intention-to-test denominator and summarizes valid-result yields, invalid/error rates (e.g., amplification failure, insufficient coverage, software errors), TATs, and repeat-test rates. Reports must distinguish callable targets (meeting depth/coverage/quality thresholds) from no-call/indeterminate and predefine handling rules to minimize bias. VAF-based interpretations should be explicit, with follow-up pDST recommended where appropriate [4,6].

Unless otherwise stated, pooled diagnostic accuracy estimates are drawn from the most comprehensive diagnostic systematic review and meta-analysis of TB tNGS to date (Schwab et al. [36]; 24 test accuracy studies). That meta-analysis applied prespecified eligibility criteria with QUADAS-2 risk-of-bias assessment and used a Bayesian bivariate random-effects model with drug- and specimen-stratified analyses and sensitivity analyses. Accordingly, pooled estimates should be interpreted in light of the between-study heterogeneity in specimens, reference standards, and assay design. Across 24 studies synthesized with a Bayesian bivariate model, tNGS showed high pooled accuracy: sensitivity: 94.1% (95% credible intervals (CrI): 90.9–96.3); specificity: 98.1% (95% CrI: 97.0–98.9). Sensitivity was ~99% for rifampicin and lower for pyrazinamide—consistent with pDST complexity and the diffuse *pncA* mutation spectrum. The accuracy was similar among specimens yielding callable targets on primary clinical specimens and culture isolates and remained robust in sensitivity analyses. Evidence was insufficient for several newer/repurposed drugs (BDQ, pretomanid, LZD, CFZ, delamanid), reflecting limited resistant isolates and incomplete variant–phenotype knowledge [36]. Accordingly, for these drugs, genotype-based calls should be interpreted strictly through WHO catalogue confidence tiers, and results should default to indeterminate in the presence of uncertain variants or insufficient coverage, triggering confirmatory pDST and/or WGS. WHO evidence summaries (Tables 2 and 3 in Web Annex D) [4] also report solution-level estimates (e.g., AmPORE-TB^®^ and DeepCheck^®^) alongside class-level tNGS estimates. These data support high overall accuracy but do not establish the unconditional superiority of any single solution across settings. Table 2 summarizes the strength of genotype–phenotype evidence by drug and the priority knowledge gaps for easier reference.

Drug-specific discordance patterns can be summarized as follows:

- Rifampicin: borderline/disrupted *rpoB* mutations—often outside the RRDR, such as I491F—can yield genotypic resistance with MICs near MGIT critical concentrations; reports should flag possible low-level resistance [37].
- Pyrazinamide: the *pncA* signal spans gene/promoter; full-gene coverage is essential, and phenotypic testing is technically challenging [38].
- FQ: most resistance maps to *gyrA* codons 90 and 94 (±*gyrB*); minor-variant calls near VAF cut-offs can explain apparent discordance and merit confirmation [6].
- LZD: *rplC* (C154R) and 23S rRNA (*rrl*) variants correlate with elevated MICs and may emerge on treatment; heteroresistance may be under-called [39].
- BDQ/CFZ axis: *Rv0678* variants frequently drive variable MIC shifts and CFZ cross-resistance; *atpE* tends to confer higher-level BDQ resistance and *pepQ* lower-level effects—together challenging current breakpoints [8,34].
- Pretomanid/delamanid: resistance involves *ddn* and F^420^ pathways (*fbiA/B/C/D, fgd1*), but critical datasets remain limited [40].

In a cross-sectional study across Georgia, India, and South Africa (*N* = 720 samples), two end-to-end tNGS workflows achieved overall complete/partial yields of 91% (Deeplex^®^ Myc-TB) and 95% (ONT TBDR^®^), rising to 96–98% in smear-positive samples. The drug-level accuracy exceeded 95% sensitivity/specificity for rifampicin and isoniazid, was high for FQs, and had ~80% sensitivity for second-line injectables and ≤50% sensitivity for LZD—emphasizing the need for stronger variant–phenotype evidence. Index test failure proportions were quantified, reinforcing predefined and follow-up rules for no-call/indeterminate outputs [41].

Operational data from South Africa and Zambia using ONT TBDR^®^ showed 66.4% success on unprocessed sputum vs. 75.0% after decontamination. The per-drug sensitivity ranged from 33% (ethionamide) to 94% (rifampicin); the specificity exceeded 90% for all drugs except CFZ. Failures clustered at low bacillary loads and when targets failed coverage thresholds, generating drug-specific no-calls [42]. These findings align with the meta-analysis and multicenter evaluation: the strongest accuracy for rifampicin/isoniazid, a consistently high performance for FQs, and variable results for some Group A/B drugs amid evolving catalogues [36,41]. On the BDQ/CFZ axis, prevalent *Rv0678* variants make genotype-positive/phenotype-borderline patterns expected; reports should pair confidence tiers with pDST follow-up [8,34].

The bacillary load (smear grade/Ct) sets the effective LoD for amplification and the depth needed to detect minor variants; yields and per-drug callability fall in paucibacillary samples, while mucolysis/decontamination and host DNA reduction can improve success [13,42]. Platform error models also matter: short reads enable lower VAF thresholds; Nanopore retains context-specific and homopolymer-associated indels despite recent gains—motivating conservative indel filters and confirmation strategies [43]. Panel design is decisive where mutation landscapes are diffuse (*pncA*) or extend beyond hotspots (*rpoB* outside RRDR); assays confined to narrow hotspots or prone to primer dropout risk sensitivity loss and no-calls [6]. Minor-variant detection claims (~1–3% in some kits) must be tuned to depth and platform errors to avoid false calls, and interpretation should track the WHO mutation catalogue [6,14,44].

The WHO’s 2025 operational handbook positions tNGS as a follow-on test for DR-TB (Algorithm 3a) to detect FQ, BDQ, and LZD resistance rapidly, enabling timely BDQ/pretomanid/LZD (BPaL) or BPaL + moxifloxacin (BPaLM) decisions; where available, other molecular tests can complement tNGS (Algorithm 3 variants) [4]. However, we emphasize that the evidence base remains limited for several newer/repurposed drugs due to sparse resistant isolate numbers and incomplete variant–phenotype mapping; therefore, BDQ/LZD/nitroimidazole-related results should be reported with confidence tiers and conservative indeterminate/reflex-testing rules. Reports should display WHO catalogue confidence tiers and link them to actions: high-confidence resistance → immediate regimen modification; uncertain/borderline findings (common for *Rv0678*) → prompt pDST and/or WGS, clearly flagging clinical risk [6,8]. Treatment initiation should not be delayed while awaiting DST; clinicians should start the best-supported regimen and update it as confirmatory results become available [45].

## 6. Linking tNGS Results to Clinical Decision Making

It is advisable to base decisions on catalogue-linked calls using predefined depth/quality and VAF thresholds, classifying each drug as susceptible/resistant/indeterminate and avoiding inference of susceptibility when coverage is inadequate. Indeterminate or discordant results generally warrant confirmatory pDST and/or WGS (Figure 1). Illustrative patient scenarios are summarized in Box 1.

Box 1 **Illustrative patient scenarios for applying tNGS calls to regimen-defining decisions (examples only). These scenarios are intended to illustrate how catalogue-linked per-drug calls (susceptible/resistant/indeterminate) and callability can be mapped to the staged approach described in Section 6. They are non-prescriptive and should be interpreted within local policy and eligibility criteria.**

Scenario 1 (straightforward, reportable across key axes): aNAAT-positive TB; tNGS reports FQ-susceptible (high confidence; callable), BDQ-susceptible (callable; no high-confidence resistance signal), and LZD-susceptible (callable). → If eligible per program criteria, initiating BPaLM without delay appears reasonable, with routine monitoring and updating if downstream pDST/WGS later contradicts the molecular result.

Scenario 2 (FQ-resistant axis): tNGS reports FQ-resistant (high confidence; callable); BDQ and LZD are susceptible and callable. → BPaL may be preferred, omitting moxifloxacin; confirmatory testing can proceed per local practice, but treatment initiation should not be delayed while adjudication is pending.

Scenario 3 (uncertain or incomplete regimen-defining calls): tNGS returns indeterminate/no-call on a regimen-defining axis (e.g., FQ no-call due to insufficient *gyrA*/*gyrB* coverage, BDQ borderline/uncertain *Rv0678* VUS/low-VAF signal, and/or LZD low-VAF signal). → Do not infer susceptibility from missing or indeterminate loci. Document uncertainty (e.g., “possible reduced susceptibility”) and trigger a predefined reflex plan (repeat tNGS on a fresh specimen or culture DNA and/or pDST/WGS). A provisional BPaLM/BPaL start with early review may be considered when benefits outweigh risks; if resistance is confirmed or early non-response/toxicity emerges, the relevant drug should be withdrawn and an individualized regimen constructed.

### 6.1. Decision Stage 1: FQ and BPaLM/BPaL Selection

- FQ-susceptible (high confidence): Initiating BPaLM without delay appears reasonable; the WHO encourages FQ-DST but notes that results should not delay BPaLM initiation [4,45,46].
- FQ-resistant or contraindicated: BPaL may be preferred, omitting moxifloxacin.
- FQ uncertain (no-call, low-confidence variant, strong clinical suspicion): A provisional BPaLM start with concurrent confirmatory testing may be considered; if resistance is confirmed, discontinuation of moxifloxacin with continuation of BPaL would be appropriate [4,45,46].

### 6.2. Decision Stage 2: BDQ Axis

- BDQ-resistant (high confidence): When catalogue-listed *atpE* substitutions or *Rv0678* Group 1/2 variants are present at the validated VAF/coverage, BPaLM/BPaL is generally avoided. Constructing an individualized, longer all-oral regimen and expediting BDQ/CFZ MIC pDST and/or WGS could be prudent [6].
- BDQ uncertain/borderline: For *Rv0678* variants of uncertain significance (VUS), low-VAF heteroresistance, or isolated *pepQ* changes, reporting “possible reduced BDQ susceptibility” may be helpful. Starting BPaLM/BPaL could be reasonable if benefits outweigh risks, with early review and confirmatory testing; if resistance is later confirmed or early non-response emerges, withdrawing BDQ would be sensible. Potential CFZ cross-resistance with *Rv0678* may be anticipated, and CFZ might best be considered not fully effective until supported phenotypically [6,47,48].

### 6.3. Decision Stage 3: LZD—Resistance and Safety

- LZD-resistant (high confidence): With canonical *rplC* (e.g., C154R) or 23S rRNA (*rrl*) domain V variants at adequate depth/VAF, BPaLM/BPaL would generally not be appropriate; an individualized, longer regimen may be warranted, with pDST/WGS for confirmation [39,46,49].
- LZD uncertain/low VAF: Reporting “possible reduced LZD susceptibility” and starting BPaLM/BPaL at the standard LZD dose with early review may be reasonable. Confirmatory testing should be considered, and discontinuation of LZD could be appropriate if resistance, non-response, or toxicity is observed. Safety management (myelosuppression, neuropathy, lactic acidosis) may follow prespecified procedures—dose reduction, interruption, or discontinuation—with therapeutic drug monitoring (TDM) considered where available [39,46,49].

### 6.4. Incomplete Outputs: No-Call, Partial Results, and Heteroresistance

- No-call (drug-specific callability failure): It would be useful for reports to indicate the reason (e.g., insufficient *gyrA* coverage); repeating tNGS on a fresh specimen or culture DNA and/or pursuing pDST/WGS may be considered.
- Partial results: Summarizing the per-drug status and indicating the regimen-decisive call (often FQ) may aid decision making.
- Heteroresistance/minor variants: Reporting the exact VAF and locus-specific LoD, labeling “possible reduced susceptibility”, and suggesting early review, confirmatory testing, or prompt resampling are advisable. The stage-specific approaches above for FQ/BDQ/LZD uncertainty may be applied [4,6].

### 6.5. Network Operations: Centralized vs. Decentralized, TAT, Informatics

A hybrid hub-and-spoke model may offer a balanced approach. Central (often Illumina-based) hubs can maximize QA/EQA and expert oversight but may incur transport-related TAT. In contrast, decentralized (often Nanopore-based) nodes could enable same-/next-day calls for priority aNAAT-positive cases, but they typically operate with smaller batch sizes and require more highly trained staff. Programs might operationalize (i) specimen logistics and run triggers (direct tNGS first; culture DNA backup), (ii) service-level targets and QC (published callability criteria, per-run controls, root-cause coding with corrective actions), and (iii) informatics/decision support—LIMS/EHR integration of computable fields (per-drug call, tier, VAF, callability, catalogue version) with clinician notifications and automated follow-up orders. Maintaining live dashboards, downtime contingencies, and governance for version control, validation, and audit would be prudent [4].

In low-resource settings, practical constraints can dominate performance in routine deployment. Workforce barriers include the limited availability of staff trained across pre-analytics, wet-lab workflows, bioinformatics, and high turnover. Programs may therefore benefit from structured competency-based training, training-of-trainers models, and centralized remote oversight. Equipment-related barriers include limited local service coverage, delays in spare parts/consumables, cold-chain interruptions, and unstable power/internet that increases downtime. Preventive maintenance plans, service-level agreements, UPS/generator contingencies, and clear hub fallback pathways can mitigate these risks. Regulatory and governance limitations (e.g., import permits for reagents/flow cells, accreditation and IVD authorization requirements, and restrictions on specimen/data transfer) can slow scale-up, supporting early engagement with national regulators and alignment with ISO-linked QA/EQA and change control processes.

### 6.6. On-Treatment Reassessment and Policy Feedback

Embedding time- and event-based triggers (non-response, microbiologic failure/reversion, toxicity, drug–drug interactions) may help keep care responsive. Each trigger could prompt resampling (preferably direct specimen), repeat tNGS, and pDST/WGS where feasible, alongside adherence review, potential TDM and dose optimization, and regimen adjustment. Feeding structured fields (e.g., per-drug calls, tiers, VAFs, callability/QC, TATs, downstream phenotypes) into dashboards may facilitate performance tracking and site-to-site comparisons. These data could support iterative updates to report templates and follow-up rules, recalibration of VAF/coverage thresholds, panel and catalogue revisions under change control, and contributions to EQA and surveillance. Regular multidisciplinary reviews may help keep the aNAAT → tNGS → WGS/pDST cascade closely aligned with outcomes [4,46].

## 7. QA, EQA, and Interpretation Software

A risk-based QA system should span pre-analytical, analytical, and post-analytical phases and align with ISO 15189 “Medical laboratories—Requirements for quality and competence” and 17025 “General requirements for the competence of testing and calibration laboratories”. Governance may include a defined system that is clearly responsible, accountable, consulted, and informed (RACI, such as laboratory director, molecular lead, bioinformatics lead, QA manager), documented report sign-off, and a complete validation package before go-live (accuracy vs. reference methods, precision, LoD and minor-variant limits, reportable range/callability criteria, contamination tolerance, interference), followed by on-site verification and a controlled dry run with LIMS integration. Ongoing QA typically maintains SOPs under change control, lot qualification of critical reagents, instrument maintenance, competency-based training, batch acceptance criteria, key performance indicator (KPI) review (valid-result yield, no-call/error rates, contamination flags, TATs, re-run frequency), corrective and preventive actions (CAPA), and audit-ready records. A formal change control process should include impact/risk assessment, orthogonal confirmation when needed, predefined retrospective re-analysis rules, and clinical communication for updates to catalogues, interpretation methods, software/containers, panels, or firmware, with linkage to EQA outcomes and management review [50,51].

Standardized acceptance/triage helps ensure that only callable inputs enter the pipeline and consists of verifying identifiers and the minimum volume; capturing the collection-to-receipt time/temperature; recording decontamination or mucolysis and inactivation; and using the smear grade or aNAAT Ct to decide between direct tNGS and culture-DNA fallback. Contamination control may include a unidirectional workflow (pre-PCR → post-PCR), physical segregation, dedicated equipment and filtered tips, surface decontamination/UV, barcode chain of custody, and lot tracking. Controls typically comprise extraction negative control, NTC, positive control with known variants, and periodic low-load challenge to monitor the LoD. Extraction QC may use fluorometric quantification, purity indices, inhibition checks, and human:Mycobacterium tuberculosis (MTB) DNA ratio thresholds. Library QC commonly evaluates the fragment size distribution, target balance, molarity/normalization, index design (dual-unique where possible), and adapter–dimer proportion, each with acceptance ranges and corrective actions. The pooling/run setup should consider reads-per-sample targets, batch size, index collision avoidance, and platform-specific run controls. Predefined fail/repeat rules can codify triggers for re-extraction, re-library, or recollection; cap repeats; escalate to pDST/WGS if criteria remain unmet; and record QC as structured fields in LIMS [50,51].

Batches are generally released only when platform-appropriate rules are satisfied or under a documented conditional-release plan. The instrument/run health (e.g., yield and % ≥Q30 for short reads; active pore count and mean Q-score for Nanopore), control checks (NTC carry-over, positive-control genotype, periodic low-load LoD tracking), and the demultiplexing quality (barcode assignment/crosstalk) should be reviewed. Post-mapping, the breadth of coverage (≥X% above depth threshold), uniformity across loci, and on-target rate are accessed, and outliers/dropouts are investigated. Contamination checks may include taxonomic screens, human:MTB ratios for direct specimens, and mixed-lineage flags. Callability rules (minimum depth/quality, strand bias, stable VAF thresholds) are applied and pipeline/software versions are recorded in the run record. Explicit batch outcomes (pass; conditional release with a predefined plan for marked no-calls; rerun; reject) and root-cause/CAPA for deviations are recommended; run KPIs can be tracked on control charts [50].

The informatics layer should enforce mapping/QC thresholds (on-target rate, alignment quality, per-locus depth/breadth, strand bias), trim/mask primer sites, and return no-call rather than over-call below thresholds. Context-aware filters (e.g., conservative indel handling in homopolymers) and bidirectional evidence for low-VAF calls are advisable: Normalize variant coordinates to a fixed reference build with standard notation and harmonize promoter conventions before catalogue lookup. Validate against a curated truth set (including synthetic heteroresistance mixes), track recall/precision by variant and drug, and run regression tests before each release. Attach the WHO TB mutation catalogue by semantic version/date and fix it in reports; maintain and update playbook (impact assessment, dual-tool cross-check, targeted re-analysis, clinician communication) to prevent catalogue drift. Report VUS/out-of-catalogue calls transparently with action text (e.g., follow-up pDST/WGS). Containerize the pipeline, distribute a reproducible bundle (commands, reference checksums, software hashes), and monitor bioinformatics KPIs with CAPA workflows [6,24].

The ELRTB-Net-2 WGS EQA model can be adapted to tNGS with two complementary arms: a wet-lab ring trial (specimen-to-report) and a dry-lab challenge (FASTQ/BAM-to-interpretation). Blinded panels should reflect the program reality: lineage diversity; archetypal resistant loci (e.g., *rpoB* including RRDR and I491F; *katG/inhA*; *pncA* full gene; *gyrA/gyrB*; *rplC/rrl*; *rrs/eis*; *Rv0678/atpE/pepQ*); low-load/host-rich specimens to stress the LoD; heteroresistance mixes (~1–5% VAF); and negative/contamination traps. Structured submissions may include per-drug call, variant lists with WHO tiers, VAFs, callability/QC, catalogue/pipeline versions, and TATs. Scoring can cover the per-drug categorical accuracy (penalizing very-major/major errors), per-target callability, variant-level concordance, report completeness, and TAT; performance bands/benchmarks may be published and CAPA-mandated for deficiencies. Annual or biannual cycles with strong governance (blinding, chain of custody, method declarations) are typical [29,52].

The Global Laboratory Initiative (GLI) EQA Dashboard can serve as the entry point to external proficiency schemes, as it is a living directory of providers/panels with filters by technology, provider, sample type/format, and contacts. A lightweight workflow—discover → enroll → plan logistics (permits/cold chain) → execute under routine conditions → ingest scorecards into LIMS → link to CAPA/competency records—supports programmatic adoption. Network-level practice may include an annual EQA calendar mapping each method (including tNGS/WGS) to a ≥1 panel, monitoring participation/pass rates, and tracking root-cause-coded non-conformances on a central QA dashboard [53].

Regarding the interpretation software, three mature tools are commonly deployed: TB-Profiler (CLI/web) ingests short or long reads (including MinION) and exports machine-readable JSON for LIMS integration; its development emphasizes rapid, automated informatics for DR-TB. Mykrobe is an offline desktop application optimized for speed and low computation with user-updatable catalogues—useful for synchronizing to WHO releases. SAM-TB is a web platform coupling QC/mapping/variant calling with genotypic DST, lineage/cluster analysis, and mixed-sample/NTM detection (e.g., via Kraken2), and it reports low-confidence mutations. Across evaluations, these tools perform robustly when catalogues are current and callability/VAF rules are standardized. Practical tips include specifying catalogue and software/container versions on every report, preferring JSON exports for interoperability, adopting a dual-tool policy for borderline calls, and scheduling re-analysis when catalogues update [24,25,54].

Head-to-head and meta-analytic assessments suggest that multiple tools perform well overall, with drug-specific differences driven by rules and training data. A systematic review and Bayesian network meta-analysis reported consistently high accuracy for TB-Profiler, Mykrobe, SAM-TB, PhyResSE, and TGS-TB (with drug-level variation), emphasizing the need for up-to-date catalogues and standardized callability/VAF thresholds. An analysis of 36,385 isolates found that the WHO 2023 catalogue yielded the highest specificity and TB-Profiler the highest sensitivity, and that an ensemble of tools improved the area under the curve (AUC)—especially for second-line drugs—supporting dual-tool policies for borderline or high-stakes calls. In practice, programs may (i) specify the WHO 2023 catalogue and software versions on every report and re-analyze priority backlogs after updates; (ii) enforce standardized callability/VAF criteria; (iii) deploy primary and secondary tools for uncertain or program-critical results (FQ/BDQ/LZD) with follow-up pDST/WGS; and (iv) integrate machine-readable outputs (JSON/HL7/FHIR) so tiers, VAFs, and versions drive auto-alerts and audit trails [55,56].

## 8. Economic and System Considerations: Cost-Effectiveness and Network Design

We summarize the adoption of tNGS as a follow-on test after the aNAAT in India, South Africa, and Georgia, focusing on the cost-effectiveness, unit cost, and network design. In multicountry modeling, assumptions about the reference strategy matter: Under current in-country DST practice, tNGS can be cost-saving or cost-effective; when universal pDST for all key drugs is assumed, pDST dominates in all three settings. A 50% reduction in kit price renders tNGS cost-effective across countries, whereas higher BDQ-resistance prevalence (~30%) reduces cost-effectiveness [5,57,58].

The case of India can be summarized as high-volume and mixed-infrastructure. Modeling suggested that relative to prevailing in-country DST practice, tNGS dominates (greater health gain at lower cost). Programmatic unit cost ranges (full-panel) are ~ USD 121–175/test, varying with volume and batching. A hub-and-spoke model is therefore attractive: state-level Illumina hubs for the lowest unit cost, complemented by regional rapid (nanopore) nodes for same-day decisions in priority aNAAT-positive cases. Where feasible, direct-specimen workflows (e.g., AmPORE-TB^®^) can return reports in ~5–6 h for ~22-sample batches; early pilots reported per-sample running costs ~ USD 230, excluding labor and capital expenditures, emphasizing the importance of throughput and batch utilization in high-volume states [5,13,58,59].

In one evaluation for South Africa, tNGS was cost-effective vs. the current in-country DST practice (incremental cost-effectiveness ratio (ICER) ~USD 15,619 per disability-adjusted life year (DALY) averted; willingness to pay (WTP) ~USD 21,165). A South Africa-specific analysis found centralized tNGS cost saving vs. standard care, while decentralized tNGS was cost-effective but more expensive per DALY due to smaller batches; the latter improved the time to a correct resistance profile and reduced the infectious time. Unit cost ranges are ~USD 134–257/test (sensitive to batching and kit price). Faster regimen selection compounds downstream value: a South African BPaL assessment estimated ~75% lower patient-incurred costs vs. longer regimens and favorable provider ICERs, implying that the earlier, accurate selection via tNGS can yield patient- and provider-side savings [5,13,57,58,60].

With ~187 eligible DR-TB cases per year, avoiding idle capacity is pivotal in Georgia. In the base case, tNGS was not cost-effective vs. the current in-country DST pathway (ICER ~USD 18,375/DALY; WTP ~USD 15,069), whereas positioning tNGS as the initial drug-resistance test was cost-effective (ICER ~USD 9261/DALY; ~80% of simulations below WTP). Unit cost ranges are ~USD 120–198/test, driven primarily by volume and batching. A centralized/hub model with explicit courier service-level agreements to maintain the network TAT appears suitable, with targeted rapid capacity only where volumes routinely support economical batches or where same-day decisions are critical [5,13,58].

Across these cases, one-way and probabilistic sensitivity analyses converge on a common set of drivers: (i) kit price, (ii) throughput/batching and capacity utilization, (iii) direct-specimen success and no-call rates (and consequent follow-up testing), and (iv) epidemiology—especially BDQ/LZD/FQ-resistance prevalence. The WHO technical brief consolidates unit cost ranges that tighten at higher volumes and shows how small run sizes inflate unit costs; conversely, platform sharing across programs improves affordability by lifting utilization. A South Africa-focused evaluation also quantifies the TAT–cost trade-off: centralized models may be cost-saving, while decentralized models improve TATs but can raise costs/testing if batch utilization falls—hence the value of explicit batching/TAT rules and event-based run triggers [5,57,58].

These findings should be interpreted as context-specific scenarios rather than universal conclusions. Key assumptions that can materially shift ICERs include (i) the local resistance prevalence (particularly along the BDQ/LZD/FQ axes), which determines how often earlier molecular results change regimen selection and downstream outcomes; (ii) the scale-up feasibility, including achievable batch sizes and capacity utilization, instrument uptime/service coverage, staffing and competency requirements, QA/EQA overhead, and whether sequencing infrastructure is shared across programs; and (iii) context-dependent comparator pathways and uptake, such as the baseline coverage of pDST, TATs in the current network, drug availability, and degree to which clinicians act on tNGS results. Because these parameters vary widely between settings and over time, programs should treat published ICERs as planning estimates and prioritize local scenario analyses using observed specimen volumes, smear/Ct distributions (affecting direct-from-specimen success/no-call rates), and current resistance surveillance data.

## 9. Discussion

This review brings together design principles, catalogue-linked interpretation, comparative workflow characteristics, accuracy evidence, QA/EQA and informatics requirements, and system economics to outline how tNGS can be implemented in practice, emphasizing what the preceding evidence implies for clinical decisions, laboratory operations, and policy.

From a care delivery perspective, the principal contribution of tNGS is expansion of the early decision window: catalogue-linked, tiered reports can clarify susceptibility to the drugs that anchor shortened all-oral regimens while leaving space for confirmation when uncertainty remains. Consistent use of coverage/VAF thresholds and confidence tiers may help prevent over-interpretation; ambiguous results are typically routed to WGS and/or pDST with clear action text in the report (e.g., early review, repeat sampling) [4,5,6]. Areas of persistent uncertainty concentrate around newer or repurposed drugs—most notably the BDQ/CFZ and nitroimidazole axes—where the catalogue breadth and phenotype linkage continue to mature [6,8].

Operational realism matters as much as analytics. For practical deployment, the tNGS implementation checklist referenced throughout this review is consolidated as a template in Box 2. The direct-from-specimen performance tends to track bacillary loads and pre-analytics, so triage rules (smear/Ct), standardized specimen handling, and transparent callability criteria are central to predictable yields; these points suggest that sites may benefit from publishing run triggers and fallback pathways [13,41,42]. On the system side, time-to-result and unit costs appear to trade off against batch size and kit price; hybrid networks that pair high-throughput hubs with rapid regional nodes offer a way to balance both, particularly when coupled to the LIMS/EHR integration of computable fields [5,57,58,59].

Box 2 **Field implementation checklist template for tNGS (specimen to report). Notes: The reportable range is the set of loci that meet prespecified depth/quality/VAF criteria in that specimen. Loci outside the range are no-call → drug indeterminate. Institutions can fill in the blank fields in the template after local validation.**

(A) Triage & specimen acceptance (direct specimen VS. culture DNA)
  - Permitted specimen types are defined (sputum; validated BALF/respiratory matrices; culture DNA).
  - Smear/acid-fast bacillus grade rule: Proceed with direct tNGS if ≥____; fallback to culture DNA if < ____.
  - aNAAT Ct rule: Direct tNGS if Ct ≤ ____; culture-DNA fallback if Ct > ____ or inhibition is suspected.
  - Host-DNA depletion and pre-analytics are specified (SOP ID: ____).
  - Transport/storage documented (collection → receipt time/temperature); minimum volume ≥____ mL; LIMS accession/barcoding enforced.
(B) Controls & contamination safeguards
  - Batch controls: Extraction-negative control, NTC, positive control (with known variants), and periodic low-load challenge included.
  - Carry-over prevention: dUTP/UNG, unidirectional workflow (pre-PCR VS. post-PCR), physical separation.
  - Index/barcode policy: Dual-unique preferred; barcode crosstalk threshold ≤____ %
(C) Library & sequencing (platform-specific)
  - Run trigger (batch size): Start a run at ≥____ samples (+ controls), or follow same-day rules for priority nodes.
  - Depth target: Median per-locus ≥20× (replace with validated site target: ≥____×).
  - Run quality checks: (Short-read) total yield, %Q30 ≥ ____ %; (nanopore) active pore ≥____, mean Q-score ≥ ____.
(D) Computational QC → callability rules
  - Trimming/masking & alignment: Primer masking; BWA-MEM2 for short reads/minimap2 (+ Medaka) for long reads.
  - Variant filters (SNP/indel): Per-locus min depth ≥____ x, base-Q ≥ ____, strand -bias limits; conservative indel handling for homopolymers.
  - Callability: Loci that fail depth/quality are outside the reportable range and become no-call (drug = indeterminate); do not infer susceptibility from missing data.
(E) VAF/LoD/LoQ (minor variants & heteroresistance)
  - Validated thresholds per locus/platform: LoD = ____ %, LoQ = ____ %; VAF < LoQ reported as “possible low-level” in narrative, not used for categorical drug calls.
  - Verification: Dilution series and synthetic mixes for heteroresistance; replicate n ≥ ____.
  - Borderline VAF policy: Trigger re-library or re-extract when ΔVAF ≤ ____ % between replicates or depth < ____ x.
(F) Interpretation & reporting (WHO catalogue-linked)
  - WHO TB mutation catalogue version/date locked (e.g., v____/YYYY-MM-DD); reports list software/container/catalogue versions.
  - Per-drug decision logic:
    1. Groups 1/2 variant present → Resistant
    2. Only Groups 4/5 and complete coverage → Susceptible
    3. Group 3/VUS, borderline VAF, or coverage failure → Indeterminate (reflex WGS/pDST)
  - BDQ/CFZ axis notes: Rv0678 signals carry CFZ cross-resistance caveat; high-confidence atpE variants support BDQ-R; isolated pepQ typically indeterminate.
  - Report layout: Gene → variant → catalogue tier → drug call, with coverage/VAF metrics and callability failure reasons
(G) Repeat/reflex rules
  - Re-extract when inhibition suspected, human:MTB ratio high, on-target <____ %.
  - Re-library for library failure/imbalance or target dropout.
  - Reflex WGS/pDST for discordant/indeterminate, borderline VAF, or clinic-epidemiologic mismatch.
  - Repeat cap: Maximum ____ repeats per specimen; beyond this, new specimen preferred.
(H) Batch release & governance
  - Control review: NTC; positive control expected variants detected; low-load challenge within LoD.
  - Explicit batch outcomes: Pass/conditional release (no-calls labelled + planned actions)/Rerun/Reject, with root-cause & CAPA.
  - EQA participation & version-lock: Pipelines/catalogues version-locked; LIMS traceability; periodic EQA/proficiency testing

Quality and governance emerge as important factors. Version-controlled wet-lab and bioinformatics SOPs, explicit callability/VAF rules, and participation in wet- and dry-lab EQA are recurrent themes across the evidence base. Interpretation software performs robustly when synchronized to current catalogues and when outputs are machine-readable; dual-tool policies may be useful for borderline or program-critical calls.

In our view, mixed infection and heteroresistance are best treated as intrinsic biological and sampling phenomena that become most difficult to resolve in culture-free tNGS at low bacillary loads and in specimen matrices with high host DNA background. Because both callability and minor-variant detectability shift with the smear grade/Ct and specimen type, a single universal “minor-variant” threshold is unlikely to be appropriate across settings. Until externally validated standards are available, it is suggested that programs define and publish performance using (i) an intention-to-test denominator with stratified valid-result yield/callability by smear/Ct and specimen type, and (ii) locus- and platform-validated LoD/LoQ and VAF rules, with conservative reporting and reflex pDST/WGS for borderline signals or suspected mixtures.

To accelerate the iterative improvement in resistance catalogues, sequencing and phenotype datasets should be deposited in long-term repositories and shared with sufficient contextual metadata. For sequence data, the International Nucleotide Sequence Database Collaboration [61] (INSDC; e.g., GenBank [62]/Sequence Read Archive (SRA) [63], European Nucleotide Archive (ENA) [64], DNA Data Bank of Japan (DDBJ) [65]) provides a widely used public infrastructure archiving nucleotide sequence data from raw reads to assembled and annotated sequences. TB-specific resources such as the National Institute of Allergy and Infectious Diseases (NIAID) TB Portals provide an open, web-based environment for sharing and analyzing de-identified multi-domain case data linking clinical/demographic information with bacterial genomic and radiological data [66]. Collaborative platforms such as Pathogenwatch allow users to organize genomes into collections that are private by default and can be shared with collaborators via a URL, supporting reproducible contextualization [67]. Where richer patient-level metadata are required, controlled-access models with formal data access governance (e.g., the database of Genotypes and Phenotypes (dbGaP) [68] or The European Genome-phenome archive (EGA) [69]) can provide proportionate safeguards; broader principles for responsible sharing may be guided by Global Alliance for Genomics & Health (GA4GH) [70] frameworks. Minimal metadata include the essential isolate context (ID, date, location, specimen/source), assay and pipeline versioning, and key DST descriptors (drug, phenotype, method/media, critical concentration or MIC) to enable reuse and genotype–phenotype linkage.

A concise forward agenda may follow from the following points: (i) externally validated performance standards for culture-free tNGS across specimen types and bacillary loads; (ii) quantitative thresholds and reporting standards for heteroresistance and mixed infection; (iii) stronger genotype–phenotype linkage for BDQ/CFZ, nitroimidazoles, and LZD; (iv) community curation and data sharing to iterate catalogues; (v) targeted validation in under-served groups (e.g., pediatric and paucibacillary disease); and (vi) standardized economic evaluations to recalibrate network designs as volumes and prices evolve [4,5,6,8,36,41,58].

Several limitations deserve note. First, catalogue-linked interpretation is inherently time-sensitive: the WHO mutation catalogue and its evidence tiers evolve as new genotype–phenotype data become available, and variant reclassification—particularly for newer/repurposed drugs—can alter per-drug calls and recommended follow-up. Second, operational and economic inputs are volatile. Reagent/kit and consumable costs, shipping and procurement conditions, and achievable batching/throughput vary by setting and over time. Therefore, the unit cost ranges and cost-effectiveness results summarized here should be interpreted as indicative rather than fixed and may shift as prices and volumes change. Third, the software ecosystem is rapidly moving (basecalling, variant calling, filtering rules, and catalogue mapping), and performance can change across software/container versions. Reproducibility therefore depends on explicit version reporting, structured change control, and predefined re-analysis triggers when catalogues or pipelines are updated. Accordingly, implementers should treat the quantitative values as planning estimates and verify them against the current manufacturer documentation and the latest WHO catalogue/software releases at the time of local validation and go-live. Finally, resistance epidemiology is dynamic and heterogeneous across regions and time. Prevalence influences predictive values and program impact, and local mutation spectra may differ from those represented in pooled estimates, particularly as drug use patterns and selective pressure change. For these reasons, this review would benefit from updating when major WHO catalogue/guideline releases occur and, in the absence of such triggers, approximately every 1–2 years as new field evaluations, kit versions, and resistance trends emerge.

## 10. Conclusions

tNGS now occupies a defined, guideline-endorsed position in TB diagnostics, enabling rapid, catalogue-linked resistance profiling across first-generation, second-generation, and newer drugs and thereby shortening the time to effective therapy while preserving space for confirmatory testing where appropriate. Real-world value appears to hinge less on novel analytics per se than on disciplined implementation: version-locked SOPs, transparent callability/VAF thresholds, EQA participation, and LIMS/EHR integration that turn results into consistent program actions.

At the service delivery level, hybrid hub-and-spoke networks may offer balanced throughput and turnaround; unit cost and callability tend to be the dominant economic drivers, with batch utilization and kit price shaping affordability. As catalogues and software continue to mature, conservative interpretations of uncertain variants—paired with clear escalation pathways—can help align speed with stewardship.

Evidence gaps remain, particularly on the BDQ/CFZ and nitroimidazole axes, heteroresistance detection limits, and standardized performance thresholds for culture-free tNGS. As these gaps narrow through the research and governance priorities summarized above, programs may apply a “right test, right time” approach with greater confidence—accelerating initial regimen decisions with tNGS and reserving WGS/pDST for adjudication and surveillance—while improving patient outcomes and safeguarding next-generation regimens across diverse settings.

## Figures and Tables

**Table 1:** Deeplex^®^ Myc-TB vs. AmPORE-TB—side-by-side comparison (illustrative implementation-oriented comparison; not exhaustive and not an endorsement).

Category	Deeplex^®^ Myc-TB (Illumina Amplicon)	AmPORE-TB^®^ (Nanopore Amplicon)
Panel content (representative loci)	Covers first-/second-line genes with tiling at highly variable regions: *rpoB, katG, inhA* promoter, *pncA, embB, gyrA/gyrB, rrs/eis, tlyA, rplC, rrl*; designed for uniformity on GC-rich/heterogeneous targets. (~18 resistance-associated genes)	Covers the same core set plus explicit nitroimidazole pathway loci (*ddn, fbiA/B/C, fgd1*) and the BDQ/CFZ axis (*Rv0678, atpE, pepQ*) for broader new-drug inference. (~24 resistance genes).
Specimen inputs (direct/culture)	Baseline: culture DNA; direct-from-sample use permissible where locally validated.	Designed for both direct specimens (e.g., sputum) and culture DNA, with triage by smear grade/aNAAT Ct and host DNA depletion as needed.
Batching/throughput	Flexible indexing supports high-throughput, centralized batching.	Rapid barcoding/pooling enables runs of ~22 samples (+controls) for small-to-mid batches suited to decentralized/rapid decisions.
Coverage/minor-variant policy	Interpretation uses locus-level depth and predefined VAF thresholds; dropouts reported as no-call → reflex (WGS/pDST) rather than inferred susceptibility.	Targets ≥20× median per locus (extend runtime if required); applies locus-specific depth/quality and conservative handling of borderline VAFs; no-call if thresholds unmet.
Typical TAT	~48 h from extracted DNA (batching/instrument dependent)	~5–6 h from extracted DNA (same-day feasibility).
Analytics/reporting/LIMS	Vendor portal performs alignment/variant calling and catalogue-linked per-drug calls, returning human-readable PDFs plus tabular exports for LIMS/EHR.	Portal pipeline adds species/lineage checks, run-QC and gene-level coverage summaries with catalogue-linked calls; outputs both narrative and tabular results for LIMS/EHR.
QA/EQA and governance	Version-locked software/catalogue and batch controls (extraction-negative; non-template control (NTC); positive control); EQA participation expected.	Same governance; for direct-specimen use, success depends on pre-analytics standard operating procedures (SOPs), triage rules, and run-level QC dashboards.
Strengths	Short-read uniformity/accuracy; strong support for high-throughput centralized workflows; mature reporting portal.	Speed (same-day) and direct-from-sample feasibility; efficient for small batches and decentralized decisions.
Limitations/cautions	TAT sensitive to batching/transport; less suitable for on-the-spot clinical decisions.	More sensitive to pre-analytics and run-time depth management; handles coverage failures/borderline VAFs conservatively.
Fit-for-purpose use	Central hubs prioritizing volume, unit cost minimization, and stable QA.	Regional/rapid nodes where same-day FQ/BDQ/LZD axis decisions are needed.

Notes: TAT and coverage figures are indicative and depend on kit version, SOPs, platform, and bacillary load.

**Table 2:** Drug-by-drug summary of genotype–phenotype evidence strength and priority knowledge gaps for TB tNGS interpretation. Evidence strength is categorized qualitatively (Strong/Moderate/Limited) using WHO mutation catalogue confidence concepts together with the synthesized diagnostic accuracy/discordance literature. The table highlights key resistance loci and the principal gaps that constrain confident molecular calls, particularly for newer/repurposed agents.

Drug	Key Loci	Evidence Strength	Key Gaps/Discordance Drivers
Rifampicin	*rpoB* (incl. outside RRDR)	Strong	Borderline/outside-RRDR variants can sit near critical concentrations → flag “possible low-level resistance” and rely on tiers/confirmation when needed.
FQ	*gyrA* (±*gyrB*)	Strong	Minor variants near VAF cut-offs can drive discordance; need validated low-VAF policies.
Pyrazinamide	*pncA* (gene/promoter)	Moderate	Diffuse mutation landscape + pDST complexity; full-gene coverage essential; better variant–MIC mapping needed.
LZD	*rplC; rrl*	Limited	Emerging on treatment; heteroresistance may be under-called; more consistent variant–MIC datasets needed.
BDQ/CFZ	*Rv0678; atpE; pepQ*	Limited	*Rv0678* has heterogeneous MIC shifts and cross-resistance; breakpoints and variant–phenotype mapping remain challenging.
Pretomanid/Delamanid	*ddn;* F^420^ pathway (*fbiA/B/C/D, fgd1*)	Limited	Critical datasets remain limited; need larger matched genotype–phenotype collections.

## Data Availability

No new data were created or analyzed in this study. Data sharing is not applicable to this article.

## Abbreviations

The following abbreviations are used in this manuscript:

aNAAT	Automated nucleic acid amplification test
AUC	Area under the curve
BALF	Bronchoalveolar lavage fluid
BDQ	Bedaquiline
CAPAs	Corrective and preventive actions
CFZ	Clofazimine
CrI	Credible interval
DALY	Disability-adjusted life year
DR-TB	Drug-resistant tuberculosis
DST	Drug susceptibility testing
dUTP	Deoxyuridine triphosphate
EHR	Electronic health record
ENA	European Nucleotide Archive
EQA	External quality assessment
FN	False negative
FP	False positive
FQ	Fluoroquinolone
GA4GH	Global Alliance for Genomics and Health
GLI	Global Laboratory Initiative
ICER	Incremental cost-effectiveness ratio
INSDC	International Nucleotide Sequence Database Collaboration
KPI	Key performance indicator
LIMS	Laboratory information management system
LoD	Limit of detection
LoQ	Limit of quantitation
LPA	Line probe assay
LZD	Linezolid
NPA	Negative percent agreement
NPV	Negative predictive value
MIC	Minimum inhibitory concentration
MTB	Mycobacterium tuberculosis
MTBC	Mycobacterium tuberculosis complex
NIAID	National Institute of Allergy and Infectious Diseases
NTC	Non-template control
NTM	Nontuberculous mycobacteria
ONT	Oxford Nanopore Technologies
OPA	Overall percent agreement
pDST	Phenotypic drug susceptibility testing
PPA	Positive percent agreement
PPV	Positive predictive value
QA	Quality assurance
QRDR	Quinolone resistance-determining region
RACI	Responsible, accountable, consulted, and informed
RRDR	Rifampicin resistance-determining region
SNP	Single-nucleotide polymorphism
SNV	Single-nucleotide variant
SOP	Standard operating procedure
SRA	Sequence Read Archive
TAT	Turnaround time
TB	Tuberculosis
TDM	Therapeutic drug monitoring
Tm	Melting temperature
TN	True negative
TP	True positive
tNGS	Targeted next-generation sequencing
UMI	Unique molecular identifier
UNG	Uracil-DNA glycosylase
VAF	Variant allele frequency
VUS	Variant of uncertain significance
WGS	Whole-genome sequencing
WHO	World Health Organization
WTP	Willingness to pay
